# Supplementary material for: Participatory learning and action (PLA) to improve health outcomes in high-income settings: a systematic review protocol
Source: BMJ Open. 2022 Feb 28;12(2):e050784. doi: 10.1136/bmjopen-2021-050784 (PMC8886484; doi:10.1136/bmjopen-2021-050784)
Supplement: Author's manuscript [file bmjopen-2021-050784.draft_revisions.pdf]

BMJ Open is committed to open peer review. As part of this commitment we make the peer review history of every article we publish publicly available.

When an article is published we post the peer reviewers' comments and the authors' responses online. We also post the versions of the paper that were used during peer review. These are the versions that the peer review comments apply to.

The versions of the paper that follow are the versions that were submitted during the peer review process. They are not the versions of record or the final published versions. They should not be cited or distributed as the published version of this manuscript.

BMJ Open is an open access journal and the full, final, typeset and author-corrected version of record of the manuscript is available on our site with no access controls, subscription charges or pay-per-view fees (http://bmjopen.bmj.com).

If you have any questions on BMJ Open's open peer review process please email info.bmjopen@bmj.com

BMJ Open

Participatory learning and action (PLA) to improve health outcomes in high income settings: Systematic Review Protocol

Journal:	*BMJ Open*
Manuscript ID	bmjopen-2021-050784
Article Type:	Protocol
Date Submitted by the Author:	28-Feb-2021
Complete List of Authors:	Allaham, Shereen ; University College London Institute of Epidemiology and Health Care, Department of Epidemiology and Public Health; Aceso Global Health Consultants Ltd,<br>Kumar, Ameeta; Royal Berkshire NHS Foundation Trust<br>Morriss, Felix; Salisbury NHS Foundation Trust<br>Manikam, Logan; University College London Institute of Epidemiology and Health Care, Department of Epidemiology and Public Health; Aceso Global Health Consultants Ltd,<br>Lakhanpaul, Monica; UCL Great Ormond Street Institute of Child Health, Population, Policy and Practice; Whittington Health NHS Trust<br>Wilson, Emma; UCL Great Ormond Street Institute of Child Health, Population, Policy and Practice<br>Sikorski, Catherine; UCL Great Ormond Street Institute of Child Health, Population, Policy and Practice<br>Martin, Jennifer; UCL Great Ormond Street Institute of Child Health, Population, Policy and Practice; Women in Global Health<br>Costello, A; UCL Great Ormond Street Institute of Child Health, Population, Policy and Practice<br>Heys, Michelle ; UCL Great Ormond Street Institute of Child Health, Population, Policy and Practice; East London NHS Foundation Trust, Specialist Children’s and Young People’s Services
Keywords:	Community child health < PAEDIATRICS, PUBLIC HEALTH, Nutrition < TROPICAL MEDICINE

SCHOLARONE™
Manuscripts

I, the Submitting Author has the right to grant and does grant on behalf of all authors of the Work (as defined in the below author licence), an exclusive licence and/or a non-exclusive licence for contributions from authors who are: i) UK Crown employees; ii) where BMJ has agreed a CC-BY licence shall apply, and/or iii) in accordance with the terms applicable for US Federal Government officers or employees acting as part of their official duties; on a worldwide, perpetual, irrevocable, royalty-free basis to BMJ Publishing Group Ltd ("BMJ") its licensees and where the relevant Journal is co-owned by BMJ to the co-owners of the Journal, to publish the Work in this journal and any other BMJ products and to exploit all rights, as set out in our licence.

The Submitting Author accepts and understands that any supply made under these terms is made by BMJ to the Submitting Author unless you are acting as an employee on behalf of your employer or a postgraduate student of an affiliated institution which is paying any applicable article publishing charge ("APC") for Open Access articles. Where the Submitting Author wishes to make the Work available on an Open Access basis (and intends to pay the relevant APC), the terms of reuse of such Open Access shall be governed by a Creative Commons licence – details of these licences and which Creative Commons licence will apply to this Work are set out in our licence referred to above.

Other than as permitted in any relevant BMJ Author's Self Archiving Policies, I confirm this Work has not been accepted for publication elsewhere, is not being considered for publication elsewhere and does not duplicate material already published. I confirm all authors consent to publication of this Work and authorise the granting of this licence.

For peer review only

Participatory learning and action (PLA) to improve health outcomes in high income settings: Systematic Review Protocol

Shereen Allaham, Research Associate[1,2]
Dr Ameeta Kumar, Foundation Doctor[3]
Dr Felix Morriss, Foundation Doctor[4]
Dr Logan Manikam, NIHR Advanced Fellow & Honorary Consultant in Public Health Medicine[1,2]
Prof Monica Lakhanpaul, Professor of integrated community child health[5,6]
Dr Emma Wilson, Research Fellow[5]
Dr Catherine Sikorski, Paediatric Registrar[5]
Jennifer Martin, Global Project Director[5,7]
Dr Anthony Costello, Professor of International Child Health and Director of the UCL Institute for Global Health[5]
Dr Michelle Heys, Associate professor of community and population child health, Consultant Paediatrician [5,8]

Corresponding author: Shereen Al Laham
Postal address: 1-19 Torrington Place, London WC1E 7HB
Email: s.laham@ucl.ac.uk

Institutions:

1. Department of Epidemiology and Public Health, University College London Institute of Epidemiology and Health Care, London, UK
2. Aceso Global Health Consultants Limited, London, UK
3. Royal Berkshire NHS Foundation Trust, Reading, UK
4. Salisbury NHS Foundation Trust, Salisbury, UK
5. Population, Policy and Practice, UCL Great Ormond Street Institute of Child Health, 30 Guildford Street, London WC1N 1EH, UK
6. Whittington Health NHS Trust, London, UK, N19 5NF
7. Women in Global Health, Washington D.C., USA
8. Specialist Children's and Young People's Services, East London NHS Foundation Trust, Newham, London, UK

For peer review only

ABSTRACT

Introduction

Participatory learning and action (PLA) is a form of group reflection and learning with documented efficacy in low-income countries (LICs) to improve health-related outcomes. PLA represents both a learning philosophy and practical framework that could be applied to a variety of contexts. To date, PLA has not been widely implemented within high-income countries (HICs) to improve health and health-related outcomes. We aim to synthesise the literature currently available by means of systematic review, to form a foundation for future applications of PLA methodology in HICs.

Methods and analysis

Two reviewers will independently search pre-defined terms in the following electronic bibliographic databases: MEDLINE, EMBASE, CINAHL and the Cochrane Library. Search terms will encompass PLA and PDSA (Plan, Do, Study Act) projects, as well as studies using the Triple/ Quadruple Aim model. We will include randomised controlled trials that incorporate online or face-to-face components using PLA/PDSA methodology. Our data will be extracted into a standardised pre-piloted form with subsequent narrative review according to Synthesis without meta-analysis (SWiM) guidelines.

Ethics and dissemination

No ethics approval is required for this study. The results for this study will be submitted for publication in a leading peer-reviewed academic journal in this field. Additionally, a report will be produced for the funders of this review, which can be viewed for free on their website.

Word count: 2576/Max 4000
PROSPERO registration number : CRD42020187978

For peer review only

STRENGTHS AND LIMITATIONS OF THIS STUDY

- Novel analysis of available evidence of PLA efficacy within HICs
- Incorporation of multiple participatory research methods to accommodate for the variation in nomenclature
- Broad inclusion criteria to allow for wide heterogeneity of PLA study designs
- Potential low number of RCTs for inclusion

INTRODUCTION

The Participatory learning and action (PLA) approach is one of many methods encompassed in the field of participatory research methods, which also include Participatory Action Research (PAR) and Community-Based Participatory Research (CBPR).[1] In comparison to traditional research methods, the underlying philosophy for these methods is to empower communities to identify their own solutions for issues that affect them, rather than for external stakeholders to enforce a solution on to them.[2,3] The onus for researchers is to seek and include diverse opinions and ensure an open space to discuss these. In doing so, researchers can gain first-hand knowledge from communities, allowing them to often collaborate with marginalised members. There are multiple techniques that researchers can use to facilitate the process, including drawing community maps of local resources or landmarks, preference rankings to prioritise issues of the group or Venn diagrams to show the sphere of influence of particular individuals or organisations.[1,3,4]

PLA group cycle is an iterative process typically led by a facilitator who facilitates the participants through a four-stage cycle of identifying and prioritizing contextual issues, designing strategies to address these issues and a post-implementation evaluation. It aims to support members to take individual and collective action to solve health problems within their community.[5] Through direct engagement, PLA serves to empower communities to understand the importance of their health and the health of their families and communities, as well as the barriers and facilitators to optimising these health outcomes. Due to its demonstrated efficacy, the World Health Organisation has recommended the implementation of groups practicing PLA in rural low income country settings with reduced access to health services in the context of maternal and newborn health.[6,7]

For peer review only

The current PLA model originated from multiple policies and theories from the last century. In his seminal work ‘Pedagogy of the Oppressed’, Paulo Freire defined that through critical evaluation, the ‘oppressed’ could re-evaluate and improve their context.[8] Around the late 1970s, Robert Chambers developed a more participatory research tool, ‘Rapid Rural Appraisal’, as an alternative to more didactic learning methods.[4] This tool later developed into the PLA cycle, but similar models have also emerged such as the Plan-Do-Study-Act (PDSA) cycle.[10] The PDSA cycle involves planning an intervention, delivering it and examining its effect before making adjustments based on feedback. As such, it has considerable overlap with the PLA model.

Within healthcare settings, PDSA cycles have been incorporated into the ‘Model for Improvement’ to expedite the Quality Improvement (QI) process.[11] QI is integral to the assessment of health services and delivering better-value, higher quality care for patients, especially important in a climate of rising healthcare costs and limited funding [12]. QI methodology can be applied to review a variety of outcomes, including waiting times, patient experience or post-operative complications. A popular framework for measuring healthcare outcomes is the ‘Triple Aim’ model.[13] Briefly, the three aims are as follows: improving patient experience of healthcare, improving health population and reducing per capita cost of healthcare. Increasingly, an additional fourth aim, to improve the wellbeing of healthcare workers, is integrated to form the ‘Quadruple Aim’.[14]

Thus far, PLA cycles have been successfully incorporated in research to improve health outcomes. Prost et al found that women’s groups that utilise PLA initiatives have reduced maternal and neonatal mortality in low-income settings (Nepal[15], India[16–18], Bangladesh[19,20] and Malawi[21,22]).[3] The most significant reduction in neonatal mortality (33%) and maternal mortality (49%) occurred in four of the sites that had the highest coverage of groups and participation of at least 30% of pregnant women. The underlying mechanisms are complex.[1,23] Some benefit can be attributed to effects such as behaviour change for hygiene, nutrition and infant care, as well as social support and birth planning. Additionally, the groups worked towards female empowerment and better decision-making to improve access to basic services, encourage safer delivery practices and reduce delays in reaching care. However, women’s groups also provide poor, excluded women a voice by building their confidence to take decisions and to reduce the stigma in their lives, while possibly reducing maternal stress.

For peer review only

PLA health initiatives in low-income countries (LICs) to date have largely focused on maternal and infant nutrition, but more recently have expanded to address both communicable[25] and non-communicable diseases[8,26]. For example, Tripathy et al set up a PLA women's group for pregnant mothers to reduce neonatal mortality in rural Jharkhand and Odisha in Eastern India.[18] For these groups, the facilitators were government-approved Accredited Social Health Activists who met with expectant or new mothers to discuss and form strategies to tackle maternal and newborn health issues. Similarly, Townsend utilised the PLA methodology to develop projects to enhance peer-based care for HIV positive prisoners in Malaysia.[25] PLA cycles have also been augmented with interventions such as health systems strengthening[22], cash transfers[27] and home counselling[28]. Harris-Fry et al combined PLA women's group with either cash or food transfers to improve maternal diets and nutrition during pregnancy in the Dhanusha and Mahottari districts in Nepal.[27] Though implementation of PLA improved diets, the addition of cash and food transfers improved the variety of diet and allocation of high energy food to pregnant mothers, respectively.

Similarly, Valdez et al. identified that youth PLA groups had a positive impact on young people in terms of substance use prevention.[24] These interventions mainly took place in the United States, with some studies included from Canada and Bosnia Herzegovina. Benefits included greater awareness of drug and alcohol misuse in their community and decreased approval of peer drug use. This was achieved partly by individual behavioural change as well as increasing youth engagement in social action. Young people were also able to gain skills in new research methods and given the opportunity to interact with key decision-makers within their community.

A phenomenon of 'global innovation', whereby innovations developed, evaluated and implemented in LIC are adapted and tested within high-income countries (HICs), is gaining popularity in HICs.[29] An exemplary initiative would be the Partners in Health's 'Prevention and Access to Care and Treatment (PACT) project. Using the home-based medical and social support service used in rural Haiti, PACT was able to increase medical adherence and therefore, positive medical outcomes for HIV patients in Boston, United States.[30] It has since been adopted across cities in the US and globally. Similarly, The Nurture Early for Optimal Nutrition (NEON) study aims to translate the achievements from Bangladesh, India, Malawi, and Nepal noted in Prost et al's work to improve infant feeding and care practices in Tower Hamlets in London.[31] Further, the co-adaptation of community participatory

For peer review only

group programmes to support and improve outcomes for parent/carers of children with neurodisability is being co-developed to be ready for piloting in Newham, East London.[32]

A further consideration for the current use of PLA is how the approach is delivered, with whom and for what outcomes and furthermore how to evaluate these approaches within HIC settings. PLA approaches within LICs are established in both online and face-to-face across all economic backgrounds. Face-to-face engagements are the norm for PLA as historically their usage has been within LICs, wherein online infrastructure tends to be less well developed. More recently, online elements have been incorporated into PLA usage in LICs. Unsurprisingly, a higher proportion of PLA applications in HICs implement online components, such as online seminars, forums and surveys for data collection.[9,33] Increased resources and improved data infrastructure enable far more rapid sharing of data and multimodal interventions as a part of PLA, whereas LIC groups cannot utilise these tools to the same extent. Equally, a comparative lack of online resources in LICs results in a predominance of face-to-face PLA groups.

As a proven cost-effective measure with the potential for significant improvement in health outcomes, there is clear applicability of PLA within high-resource settings. Studies to date have variously centred upon the viability and structure of PLA groups in high and low-resource settings, involving multiple populations and contexts.[34,35] At present, no systematic review exists, which synthesises the populations, types of intervention (online or face-to-face) and specific health-related outcomes of PLA groups in HICs.

In this review, we aim to identify the number and types of communities that have engaged in PLA methodology in high-resource settings to date and to analyse any existing documented outcomes of PLA for health and social determinants of health.

METHODS AND ANALYSIS

This protocol was developed in accordance with the Preferred Reporting Items for Systematic Reviews and Meta-Analyses Protocols (PRISMA-P) checklist [36]. This protocol has been registered in PROSPERO (CRD42020187978).

Population, interventions and outcomes

For peer review only

The review will examine published studies which assess the beneficial effects of PLA groups in social and health outcomes. Interventions can include online or face-to-face groups based in HICs, classified according to the World Bank classification as countries with an income per capita of greater than $12,535.[37] The primary outcome would be any reported improvement related to health or social issues after incorporating the PLA model.

Inclusion and exclusion criteria

We will include randomised controlled trials (RCTs) in HICs that incorporate online or face-to-face group sessions, which apply the learning and action framework. The review will comprise articles published between 2000 and 2020, and exclude any article not written in English.

Aside from participatory action research, we will incorporate studies that incorporate the PDSA and Triple/ Quadruple aim frameworks.

Search strategy

Two reviewers will independently search pre-defined terms in the following electronic bibliographic databases: MEDLINE, EMBASE, CINAHL and the Cochrane Library. Search terms will encompass PLA and PDSA projects, as well as studies using the Triple/ Quadruple Aim model (Table 1).

Table 1 Proposed terms for search strategy

**Population**	High-income settings filter
**Intervention**	“action learning" or "active learning" or "action cycle" or "learning cycle" or "experiential learning" or "problem-based learning" or “peer support” or “telephone support” or “home visits” “online support group” or “support group” or “participatory group” or “online participatory group” or “participatory learning and action” or “participatory action” or “community participation” or “PDSA” or “PDCA” or “plan-do-study-act” or “plan do study act” or “plan-do-check-act” or “plan do check act” or “women’s group*” or “quality improv*” or “quality enhanc*”
**Outcomes**	“triple aim” or “quadruple aim” or “satisfaction” or “patient experience” or “experience of care” or “quality of life” or “health outcome” or “population health” or “per capita” or “cost” or “cost-effectiveness” or “expenditure” or “cost analysis”

Data analysis and synthesis

Abstracts and articles retrieved from the databases will be imported into the EndNote reference management. After removing duplicates, two independent

For peer review only

authors will review the search results to identify those meeting the inclusion criteria. The first stage will screen the studies using only the title and abstracts before proceeding to review the full texts. Our preliminary search indicated that there is little literature available on community groups propagating a PLA cycle to improve specific health and social outcomes in high-income countries. Therefore, we will take care to compare studies with disparate aims. A flow chart will be created to show an overview of the selection process, including the number of excluded papers and the reasons for exclusion (Figure 1). At any stage, if there are any disagreements between the authors, a third reviewer will be involved to resolve it through discussion.

Data will be extracted independently by two reviewers using a standardised, pre-piloted form. The extracted information will include: name of the study, study setting, publication date, journal, authors, sample size, study population, participant demographics and baseline characteristics, population inclusion criteria, details of intervention and control, study methodology, recruitment and study completion rates, length of study, follow-up retention percentage, age of participants, outcomes and times of measurement, indicators of acceptability to users, suggested mechanisms of intervention action, RR, mean differences, type of data analysis, information for risk of bias assessment, source of funding and conflict of interest. Any discrepancies will be resolved through discussion or if necessary, a third reviewer. Missing data will be requested by contacting study authors.

Since we anticipate the outcome of selected studies to vary significantly, we will provide a narrative synthesis of the findings from the included studies without conducting a quantitative analysis. The narrative synthesis will be reported according to the SwiM guidelines and will address the methodology of each study, including assessing for risk of bias.[38] Included studies will be grouped according to the type of intervention, target population characteristics, type of outcome and intervention content.

Due to the expected diverse nature of interventions, we will use a standardised metric to represent the result of each outcome. We will calculate risk ratios for dichotomous outcomes and standardised mean differences for continuous outcomes. The results of each study will be summarised and presented in a tabular form. In doing so, we can systematically assess each study result and assess heterogeneity of key study characteristics. If sufficient data is available, we will conduct a subgroup analysis of different types of participants, for example, women or children.

For peer review only

Assessment of risk of bias

Two reviewers will independently assess the risk of bias in included studies by considering the Cochrane Collaboration's tool.[39] We will appraise the following characteristics: random sequence generation, allocation concealment, blinding of participants, personnel and outcome assessment, completeness of outcome data, selective outcome reporting and any other potential sources of bias. The reviewers will assess each domain as low, unclear or high risk as per the tool. Any disparities in the assessment of risk between the reviewers will be resolved through discussion, involving a third reviewer where necessary.

DISCUSSION

Following preliminary search, a number of trials exist describing action learning or similar interventions with patient groups in HICs to improve health and social outcomes [40-54]. PLA is a cost-effective and context specific model which has been shown to improve health outcomes in LICs. Therefore, there exists a need to clarify how the field can advance in its understanding of current documented applications of PLA within HIC settings.

We aim to bridge this gap by conducting a systematic review of the current available evidence on PLA in HICs, in order to understand present implementation and better inform future trials. We anticipate a broad heterogeneity in the data gathered owing to the wide contextuality of PLA and the flexibility of recorded outcomes under the PLA philosophy.

Inclusion of both RCTs and trials utilising the PDSA cycle/PLA groups will address direct and indirect usage of PLA and account for all documented use.

We hope that the findings of our review will result in the following outcomes. First, to establish a benchmark in the field for future reference. Second, the ability to elaborate or clarify existing applications of PLA to replicate observed effects. Third, to identify health outcomes that have not previously received attention with regards to implementation of PLA methodology in any setting. Fourth, to examine which PLA modalities relate to which health outcomes and evaluate the significance of these.

We also hope that our review will inform the designing and planning phases of future trials. This could be through guiding possible effect and therefore

For peer review only

sample sizes, selecting appropriate outcomes and suitable follow-up periods. As it has within several LICs, implementation of PLA could pave the way to influencing healthcare policies and inducing systemic changes to health provision. In summary, a comprehensive review of current literature will enable prioritisation of future research directions.

ETHICS AND DISSEMINATION

No ethics approval is required for this study. The results for this study will be submitted for publication in a leading peer-reviewed academic journal in this field. Additionally, a report will be produced for the funders of this review, which can be viewed for free on their website.

For peer review only

References

1. de Brún T, O'Reilly-De Brún M, Van Weel-Baumgarten E, *et al.* Using Participatory Learning & Action (PLA) research techniques for inter-stakeholder dialogue in primary healthcare: an analysis of stakeholders' experiences. *Research Involvement and Engagement* 2017;**3**:28. doi:10.1186/s40900-017-0077-8
2. Intrac. Participatory Learning and Action (PLA). 2017. https://www.intrac.org/wpcms/wp-content/uploads/2017/01/Participatory-learning-and-action.pdf (accessed 4 Nov 2020).
3. Chambers R. Relaxed and Participatory Appraisal: notes on practical approaches and methods for participants in PRA/PLA-related familiarisation workshops. Participation at Resource Centre, Institute of Development Studies, Brighton, UK. 2002.http://eepsea.org/uploads/user-S/11491553671Reader_1_PRA_notes.pdf (accessed 8 Nov 2020).
4. Appel K, Buckingham E, Jodoin K, *et al.* Participatory Learning and Action Toolkit: For Application in BSR's Global Programs. *Bsr* 2012;:48.https://herproject.org/files/toolkits/HERproject-Participatory-Learning.pdf (accessed 4 Nov 2020).
5. Seward N, Neuman M, Colbourn T, *et al.* Effects of women's groups practising participatory learning and action on preventive and care-seeking behaviours to reduce neonatal mortality: A meta-analysis of cluster-randomised trials. *PLoS medicine* 2017;**14**:e1002467. doi:10.1371/journal.pmed.1002467
6. World Health Organization. WHO recommendation on facilitated participatory learning and action cycles with women`s groups during pregnancy. WHO Reproductive Health Library. 2016.https://extranet.who.int/rhl/topics/improving-health-system-performance/implementation-strategies/who-recommendation-facilitated-participatory-learning-and-action-cycles-womens-groups-during (accessed 21 May 2020).
7. Prost A, Colbourn T, Seward N, *et al.* Women's groups practising participatory learning and action to improve maternal and newborn health in low-resource settings: A systematic review and meta-analysis. *The Lancet* 2013;**381**:1736–46.

doi:10.1016/S0140-6736(13)60685-6

8. Morrison J, Akter K, Jennings HM, *et al.* Participatory learning and action to address type 2 diabetes in rural Bangladesh: A qualitative process evaluation. *BMC Endocrine Disorders* 2019;**19**. doi:10.1186/s12902-019-0447-3
9. Cueva K, Cueva M, Revels L, *et al.* A Framework for Culturally Relevant Online Learning: Lessons from Alaska's Tribal Health Workers. *Journal of Cancer Education* 2019;**34**:647–53. doi:10.1007/s13187-018-1350-8
10. Deming W. *The New Economics for Industry, Government, Education (2nd edition) | IHI - Institute for Healthcare Improvement*. Cambridge, Massachusetts: : The MIT Press 2000. http://www.ihi.org/resources/Pages/Publications/NewEconomicsforIndustryGovernmentEducation.aspx (accessed 23 Aug 2020).
11. Institute of Healthcare Improvement. Science of Improvement: How to Improve. Institute of Healthcare Improvement. 2009.http://www.ihi.org/resources/Pages/HowtoImprove/ScienceofImprovementHowtoImprove.aspx (accessed 23 Aug 2020).
12. Jones B, Vaux E, Olsson-Brown A. How to get started in quality improvement. *BMJ (Online)* 2019;**364**. doi:10.1136/bmj.k5437
13. Berwick DM, Nolan TW, Whittington J. The triple aim: Care, health, and cost. *Health Affairs* 2008;**27**:759–69. doi:10.1377/hlthaff.27.3.759
14. Sikka R, Morath JM, Leape L. The quadruple aim: Care, health, cost and meaning in work. *BMJ Quality and Safety* 2015;**24**:608–10. doi:10.1136/bmjqs-2015-004160
15. Manandhar PDS, Osrin D, Prasad Shrestha B, *et al.* Effect of a participatory intervention with women's groups on birth outcomes in Nepal: Cluster-randomised controlled trial. *Lancet* 2004;**364**:970–9. doi:10.1016/S0140-6736(04)17021-9
16. Tripathy P, Nair N, Barnett S, *et al.* Effect of a participatory intervention with women's groups on birth outcomes and maternal depression in Jharkhand and Orissa, India: a cluster-randomised controlled trial. *The Lancet* 2010;**375**:1182–92. doi:10.1016/S0140-6736(09)62042-0
17. More NS, Bapat U, Das S, *et al.* Community mobilization in Mumbai slums to improve perinatal care and outcomes: A cluster randomized controlled trial. *PLoS Medicine* 2012;**9**:e1001257. doi:10.1371/journal.pmed.1001257
18. Tripathy P, Nair N, Sinha R, *et al.* Effect of participatory women's groups facilitated by Accredited Social Health Activists on birth outcomes in rural eastern India: A cluster-randomised controlled trial. *The Lancet Global Health* 2016;**4**:e119–28. doi:10.1016/S2214-109X(15)00287-9
19. Azad K, Barnett S, Banerjee B, *et al.* Effect of scaling up women's groups on birth outcomes in three rural districts in Bangladesh: a cluster-randomised controlled trial. *The Lancet* 2010;**375**:1193–202. doi:10.1016/S0140-6736(10)60142-0
20. Fottrell E, Azad K, Kuddus A, *et al.* The effect of increased coverage of participatory women's groups on neonatal mortality in Bangladesh: A cluster randomized trial. *JAMA Pediatrics* 2013;**167**:816–25. doi:10.1001/jamapediatrics.2013.2534
21. Lewycka S, Mwansambo C, Kazembe P, *et al.* A cluster randomised controlled trial of the community effectiveness of two interventions in rural Malawi to improve health care and to reduce maternal, newborn and infant mortality. *Trials* 2010;**11**. doi:10.1186/1745-6215-11-88
22. Colbourn T, Nambiar B, Bondo A, *et al.* Effects of quality improvement in health

For peer review only

facilities and community mobilization through women's groups on maternal, neonatal and perinatal mortality in three districts of Malawi: MaiKhanda, a cluster randomized controlled effectiveness trial. *International Health* 2013;**5**:180–95. doi:10.1093/inthealth/iht011

23. Gram L, Skordis-Worrall J, Manandhar DS, *et al.* The long-term impact of community mobilisation through participatory women's groups on women's agency in the household: A follow-up study to the Makwanpur trial. *PLoS ONE* 2018;**13**. doi:10.1371/journal.pone.0197426
24. Valdez ES, Skobic I, Valdez L, *et al.* Youth Participatory Action Research for Youth Substance Use Prevention: A Systematic Review. *Substance Use and Misuse* 2020;**55**:314–28. doi:10.1080/10826084.2019.1668014
25. Townsend D. Prisoners with HIV/AIDS: A participatory learning and action initiative in Malaysia. *Tropical Doctor* 2001;**31**:8–10. doi:10.1177/004947550103100103
26. Fottrell E, Ahmed N, Morrison J, *et al.* Community groups or mobile phone messaging to prevent and control type 2 diabetes and intermediate hyperglycaemia in Bangladesh (DMagic): a cluster-randomised controlled trial. *The Lancet Diabetes and Endocrinology* 2019;**7**:200–12. doi:10.1016/S2213-8587(19)30001-4
27. Harris-Fry HA, Paudel P, Harrisson T, *et al.* Participatory women's groups with cash transfers can increase dietary diversity and micronutrient adequacy during pregnancy, whereas women's groups with food transfers can increase equity in intrahousehold energy allocation. *Journal of Nutrition* 2018;**148**:1472–83. doi:10.1093/jn/nxy109
28. Nair N, Tripathy P, Sachdev HS, *et al.* Effect of participatory women's groups and counselling through home visits on children's linear growth in rural eastern India (CARING trial): a cluster-randomised controlled trial. *The Lancet Global Health* 2017;**5**:e1004–16. doi:10.1016/S2214-109X(17)30339-X
29. DePasse JW, Lee PT. A model for 'reverse innovation' in health care. *Globalization and Health* 2013;**9**:40. doi:10.1186/1744-8603-9-40
30. Behforouz HL, Farmer PE, Mukherjee JS. From directly observed therapy to Accompagnateurs: Enhancing AIDS treatment outcomes in Haiti and in Boston. *Clinical Infectious Diseases* 2004;**38**:S429–36. doi:10.1086/421408
31. Lakhanpaul M, Benton L, Lloyd-Houldey O, *et al.* Nurture Early for Optimal Nutrition (NEON) programme: qualitative study of drivers of infant feeding and care practices in a British-Bangladeshi population. *BMJ open* 2020;**10**:e035347. doi:10.1136/bmjopen-2019-035347
32. Skopec M, Issa H, Harris M. Delivering cost effective healthcare through reverse innovation. *The BMJ* 2019;**367**:l6205. doi:10.1136/bmj.l6205
33. Ambrose M, Murray L, Handoyo NE, *et al.* Learning global health: a pilot study of an online collaborative intercultural peer group activity involving medical students in Australia and Indonesia. BMC Medical Education 2017;17:1–11. doi:10.1186/s12909-016-0851-6
34. Anyon Y, Bender K, Kennedy H, et al. A Systematic Review of Youth Participatory Action Research (YPAR) in the United States: Methodologies, Youth Outcomes, and Future Directions. Health Education and Behavior 2018;45:865–78. doi:10.1177/1090198118769357
35. Backhouse T, Kenkmann A, Lane K, et al. Older Care-Home residents as collaborators or advisors in research: A systematic review. Age and Ageing 2016;45:337–45.

For peer review only

doi:10.1093/ageing/afv201
36. Moher D, Shamseer L, Clarke M, Ghersi D, Liberati A, Petticrew M, Shekelle P, Stewart LA. Preferred Reporting Items for Systematic Review and Meta-Analysis Protocols (PRISMA-P) 2015 statement. Syst Rev. 2015;4(1):1.
37. World Bank Data Team. New country classifications by income level: 2019-2020. World Bank Blogs. 2019.https://blogs.worldbank.org/opendata/new-country-classifications-income-level-2019-2020 (accessed 22 June 2020).
38. Campbell M, McKenzie JE, Sowden A, et al. Synthesis without meta-analysis (SWiM) in systematic reviews: Reporting guideline. The BMJ 2020;368. doi:10.1136/bmj.l6890
39. Sterne JAC, Savović J, Page MJ, et al. RoB 2: A revised tool for assessing risk of bias in randomised trials. The BMJ 2019;366. doi:10.1136/bmj.l4898
40. Anderson RM, Funnell MM, Nwankwo R, Gillard ML, Oh M, Fitzgerald JT. Evaluating a problem-based empowerment program for African Americans with diabetes: results of a randomized controlled trial. Ethnicity & disease [Internet]. 2005; 15(4):[671-8 pp.]. Available from: http://onlinelibrary.wiley.com/o/cochrane/clcentral/articles/745/CN-00552745/frame.html.
41. Drysdale J, Casey J, Porter-Armstrong A. Effectiveness of training on the community skills of children with intellectual disabilities. Scandinavian Journal of Occupational Therapy. 2008;15(4):247-55. PubMed PMID: 18855229.
42. Jacobsson LR, Friedrichsen M, Goransson A, Hallert C. Impact of an active patient education program on gastrointestinal symptoms in women with celiac disease following a gluten-free diet: a randomized controlled trial. Gastroenterology Nursing. 2012;35(3):200-6. PubMed PMID: 22647800.
43. Kamwendo K, Tingström P, Bergdahl B, Svensson E. Effect of problem-based learning on stages of change for exercise behaviour in patients with coronary artery disease. Physiotherapy research international : the journal for researchers and clinicians in physical therapy [Internet]. 2004; 9(1):[24-32 pp.]. Available from: http://onlinelibrary.wiley.com/o/cochrane/clcentral/articles/914/CN-00467914/frame.html.
44. Ringsberg KC, Timpka T. Clinical health education for patients with asthma-like symptoms but negative asthma tests. Allergy [Internet]. 2001; 56(11):[1049-54 pp.]. Available from: http://onlinelibrary.wiley.com/o/cochrane/clcentral/articles/298/CN-00375298/frame.html.
45. Tingström PR, Ekelund U, Kamwendo K, Bergdahl B. Effects of a problem-based learning rehabilitation program on physical activity in patients with coronary artery disease. Journal of cardiopulmonary rehabilitation [Internet]. 2006; 26(1):[32-8 pp.]. Available from: http://onlinelibrary.wiley.com/o/cochrane/clcentral/articles/237/CN-00564237/frame.html.
46. Naik AD, Teal CR, Rodriguez E, Haidet P. Knowing the ABCs: a comparative effectiveness study of two methods of diabetes education. Patient education and counseling [Internet]. 2011; 85(3):[383-9 pp.]. Available from: http://onlinelibrary.wiley.com/o/cochrane/clcentral/articles/479/CN-00837479/frame.html.

For peer review only

47. Nunez M, Nunez E, Yoldi C, Quinto L, Hernandez Ma V, Munoz-Gomez J. Health-related quality of life in rheumatoid arthritis: Therapeutic education plus pharmacological treatment versus pharmacological treatment only. Rheumatology International. 2006 June;26(8):752-7. PubMed PMID: 2006231426.
48. Nunez M, Nunez E, Segur JM, Macule F, Quinto L, Hernandez MV, et al. The effect of an educational program to improve health-related quality of life in patients with osteoarthritis on waiting list for total knee replacement: A randomized study. Osteoarthritis and Cartilage. 2006 March;14(3):279-85. PubMed PMID: 2006107634.
49. Pichert JW, Snyder GM, Kinzer CK, Boswell EJ. Problem solving anchored instruction about sick days for adolescents with diabetes. Patient Education & Counseling. 1994;23(2):115-24. PubMed PMID: 21207910.
50. Rosen L, Zucker D, Brody D, Engelhard D, Manor O. The effect of a handwashing intervention on preschool educator beliefs, attitudes, knowledge and self-efficacy. Health education research. 2009;24(4):686-98. PubMed PMID: 19318523.
51. Sarkadi A, Rosenqvist U. Experience-based group education in Type 2 diabetes: a randomised controlled trial. Patient Education & Counseling. 2004;53(3):291-8. PubMed PMID: 15186866.
52. Wong MCS, Lau TCM, Lee A. The Impact of Leadership Programme on Self-Esteem and Self-Efficacy in School: A Randomized Controlled Trial. PLoS ONE. 2012 18 Dec;7(12). PubMed PMID: 2012742201.
53. Ring Jacobsson L, Friedrichsen M, Goransson A, Hallert C. Does a Coeliac School increase psychological well-being in women suffering from coeliac disease, living on a gluten-free diet? Journal of Clinical Nursing. 2012;21(5-6):766-75. PubMed PMID: 22039932.
54. Reid MJ, Webster-Stratton C, Hammond M. Enhancing a classroom social competence and problem-solving curriculum by offering parent training to families of moderate- to high-risk elementary school children. Journal of Clinical Child & Adolescent Psychology. 2007;36(4):605-20. PubMed PMID: 18088218.

For peer review only

Authors' statement:

MH, SA, LM, ML conceived the original concept of the study and designed the research methodology. SA, AK, and FM carried out the registration and drafted the manuscript. MH, ML, LM, EW, CS, JM, and AC validated the study and revised the manuscript critically for important intellectual content. SA, AK, FM contributed to the manuscript writing, edited the final manuscript and prepared it for submission. MH, SA had primary responsibility for the final content. All authors read and contributed to reviewing the paper, the designing of the manuscript, and the approval of the final manuscript.

Funding statement:

Logan Manikam and Shereen Al Laham are funded via a National Institute for Health Research (NIHR) Advanced Fellowship (Ref: NIHR300020) to undertake the pilot randomised controlled trial of the NEON study in East London. Dr Michelle Heys is funded by Barts Charity to

undertake G2KCP study. Prof Monica Lakhanpaul was funded by the NIHR Collaboration for Leadership in Applied Health Research and Care (CLAHRC) North Thames.

Conflicts of interests statement:
There is no conflict of interest to declare.

Data sharing and availability statement:
The data from this study is available from the corresponding author upon reasonable request.

Patient and public involvement statement:
It was not appropriate or possible to involve patients or the public in the design, or conduct, or reporting, or dissemination plans of our research.

For peer review only

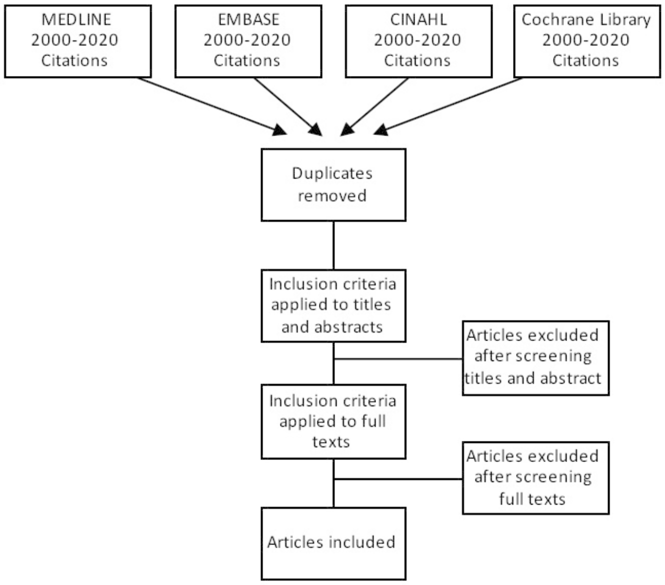

Preferred Reporting Items for Systematic Reviews- flow diagram of the search selection for this systematic review.

Reporting checklist for protocol of a systematic review and meta analysis.

Based on the PRISMA-P guidelines.

Instructions to authors

Complete this checklist by entering the page numbers from your manuscript where readers will find each of the items listed below.

Your article may not currently address all the items on the checklist. Please modify your text to include the missing information. If you are certain that an item does not apply, please write "n/a" and provide a short explanation.

Upload your completed checklist as an extra file when you submit to a journal.

In your methods section, say that you used the PRISMA-Preporting guidelines, and cite them as:

Moher D, Shamseer L, Clarke M, Ghersi D, Liberati A, Petticrew M, Shekelle P, Stewart LA. Preferred Reporting Items for Systematic Review and Meta-Analysis Protocols (PRISMA-P) 2015 statement. Syst Rev. 2015;4(1):1.

For peer review only

		Reporting Item	Page Number
**Title**			
Identification	#1a	Identify the report as a protocol of a systematic review	1
Update	#1b	If the protocol is for an update of a previous systematic review, identify as such	N/A - not for an update of a previous systematic review
**Registration**			
	#2	If registered, provide the name of the registry (such as PROSPERO) and registration number	2
**Authors**			
Contact	#3a	Provide name, institutional affiliation, e-mail address of all protocol authors; provide physical mailing address of corresponding author	1

Contribution	#3b	Describe contributions of protocol authors and identify the guarantor of the review	15
**Amendments**			
	#4	If the protocol represents an amendment of a previously completed or published protocol, identify as such and list changes; otherwise, state plan for documenting important protocol amendments	N/A - not an amendment of a previous protocol
**Support**			
Sources	#5a	Indicate sources of financial or other support for the review	15
Sponsor	#5b	Provide name for the review funder and / or sponsor	15
Role of sponsor or funder	#5c	Describe roles of funder(s), sponsor(s), and / or institution(s), if any, in developing the protocol	15
**Introduction**			
Rationale	#6	Describe the rationale for the review in the context of what is already known	3-6
Objectives	#7	Provide an explicit statement of the question(s) the review will address with reference to participants, interventions, comparators, and outcomes (PICO)	6,7
**Methods**			
Eligibility criteria	#8	Specify the study characteristics (such as PICO, study design, setting, time frame) and report characteristics (such as years considered, language, publication status) to be used as criteria for eligibility for the review	7
Information sources	#9	Describe all intended information sources (such as electronic databases, contact with study authors, trial registers or other grey literature sources) with planned dates of coverage	7
Search strategy	#10	Present draft of search strategy to be used for at least one electronic database, including planned limits, such that it could be repeated	7,8

For peer review only

Study records - data management	#11a	Describe the mechanism(s) that will be used to manage records and data throughout the review	7,8
Study records - selection process	#11b	State the process that will be used for selecting studies (such as two independent reviewers) through each phase of the review (that is, screening, eligibility and inclusion in meta-analysis)	7,8
Study records - data collection process	#11c	Describe planned method of extracting data from reports (such as piloting forms, done independently, in duplicate), any processes for obtaining and confirming data from investigators	7,8
Data items	#12	List and define all variables for which data will be sought (such as PICO items, funding sources), any pre-planned data assumptions and simplifications	6-8
Outcomes and prioritization	#13	List and define all outcomes for which data will be sought, including prioritization of main and additional outcomes, with rationale	6-8
Risk of bias in individual studies	#14	Describe anticipated methods for assessing risk of bias of individual studies, including whether this will be done at the outcome or study level, or both; state how this information will be used in data synthesis	9
Data synthesis	#15a	Describe criteria under which study data will be quantitatively synthesised	N/A - quantitative synthesis
Data synthesis	#15b	If data are appropriate for quantitative synthesis, describe planned summary measures, methods of handling data and methods of combining data from studies, including any planned exploration of consistency (such as I2, Kendall's τ)	9
Data synthesis	#15c	Describe any proposed additional analyses (such as sensitivity or subgroup analyses, meta-regression)	9
Data synthesis	#15d	If quantitative synthesis is not appropriate, describe the type of summary planned	9
Meta-bias(es)	#16	Specify any planned assessment of meta-bias(es) (such as publication bias across studies, selective reporting within studies)	9

For peer review only

Confidence in cumulative evidence	#17	Describe how the strength of the body of evidence will be assessed (such as GRADE)	9

Notes:

- 1b: N/A - not for an update of a previous systematic review
- 4: N/A - not an amendment of a previous protocol
- 15a: N/A - quantitative synthesis The PRISMA-P elaboration and explanation paper is distributed under the terms of the Creative Commons Attribution License CC-BY. This checklist was completed on 28. February 2021 using https://www.goodreports.org/, a tool made by the EQUATOR Network in collaboration with Penelope.ai

For peer review only

BMJ Open

Participatory learning and action (PLA) to improve health outcomes in high income settings: Systematic Review Protocol

Journal:	*BMJ Open*
Manuscript ID	bmjopen-2021-050784.R1
Article Type:	Protocol
Date Submitted by the Author:	08-Sep-2021
Complete List of Authors:	Allaham, Shereen ; University College London Institute of Epidemiology and Health Care, Department of Epidemiology and Public Health; Aceso Global Health Consultants Ltd,<br>Kumar, Ameeta; Royal Berkshire NHS Foundation Trust<br>Morriss, Felix; Salisbury NHS Foundation Trust<br>Manikam, Logan; University College London Institute of Epidemiology and Health Care, Department of Epidemiology and Public Health; Aceso Global Health Consultants Ltd,<br>Lakhanpaul, Monica; UCL Great Ormond Street Institute of Child Health, Population, Policy and Practice; Whittington Health NHS Trust<br>Wilson, Emma; UCL Great Ormond Street Institute of Child Health, Population, Policy and Practice<br>Sikorski, Catherine; UCL Great Ormond Street Institute of Child Health, Population, Policy and Practice<br>Martin, Jennifer; UCL Great Ormond Street Institute of Child Health, Population, Policy and Practice; Women in Global Health<br>Costello, A; UCL Great Ormond Street Institute of Child Health, Population, Policy and Practice<br>Heys, Michelle ; UCL Great Ormond Street Institute of Child Health, Population, Policy and Practice; East London NHS Foundation Trust, Specialist Children’s and Young People’s Services
**Primary Subject Heading**:	Public health
Secondary Subject Heading:	Public health
Keywords:	Community child health < PAEDIATRICS, PUBLIC HEALTH, Nutrition < TROPICAL MEDICINE

SCHOLARONE™
Manuscripts

I, the Submitting Author has the right to grant and does grant on behalf of all authors of the Work (as defined in the below author licence), an exclusive licence and/or a non-exclusive licence for contributions from authors who are: i) UK Crown employees; ii) where BMJ has agreed a CC-BY licence shall apply, and/or iii) in accordance with the terms applicable for US Federal Government officers or employees acting as part of their official duties; on a worldwide, perpetual, irrevocable, royalty-free basis to BMJ Publishing Group Ltd ("BMJ") its licensees and where the relevant Journal is co-owned by BMJ to the co-owners of the Journal, to publish the Work in this journal and any other BMJ products and to exploit all rights, as set out in our licence.

The Submitting Author accepts and understands that any supply made under these terms is made by BMJ to the Submitting Author unless you are acting as an employee on behalf of your employer or a postgraduate student of an affiliated institution which is paying any applicable article publishing charge ("APC") for Open Access articles. Where the Submitting Author wishes to make the Work available on an Open Access basis (and intends to pay the relevant APC), the terms of reuse of such Open Access shall be governed by a Creative Commons licence – details of these licences and which Creative Commons licence will apply to this Work are set out in our licence referred to above.

Other than as permitted in any relevant BMJ Author's Self Archiving Policies, I confirm this Work has not been accepted for publication elsewhere, is not being considered for publication elsewhere and does not duplicate material already published. I confirm all authors consent to publication of this Work and authorise the granting of this licence.

For peer review only

Participatory learning and action (PLA) to improve health outcomes in high income settings: Systematic Review Protocol

Shereen Allaham, Research Associate[1,2]
Dr Ameeta Kumar, Foundation Doctor[3]
Dr Felix Morriss, Foundation Doctor[4]
Dr Logan Manikam, NIHR Advanced Fellow & Honorary Consultant in Public Health Medicine[1,2]
Prof Monica Lakhanpaul, Professor of integrated community child health[5,6]
Dr Emma Wilson, Research Fellow[5]
Dr Catherine Sikorski, Paediatric Registrar[5]
Jennifer Martin, Global Project Director[5,7]
Dr Anthony Costello, Professor of International Child Health and Director of the UCL Institute for Global Health[5]
Dr Michelle Heys, Associate professor of community and population child health, Consultant Paediatrician [5,8]

Corresponding author: Shereen Al Laham
Postal address: 1-19 Torrington Place, London WC1E 7HB
Email: s.laham@ucl.ac.uk

Institutions:

1. Department of Epidemiology and Public Health, University College London Institute of Epidemiology and Health Care, London, UK
2. Aceso Global Health Consultants Limited, London, UK
3. Royal Berkshire NHS Foundation Trust, Reading, UK
4. Salisbury NHS Foundation Trust, Salisbury, UK
5. Population, Policy and Practice, UCL Great Ormond Street Institute of Child Health, 30 Guildford Street, London WC1N 1EH, UK
6. Whittington Health NHS Trust, London, UK, N19 5NF
7. Women in Global Health, Washington D.C., USA
8. Specialist Children's and Young People's Services, East London NHS Foundation Trust, Newham, London, UK

For peer review only

ABSTRACT

Introduction

Participatory learning and action (PLA) is a form of group reflection and learning with documented efficacy in low-income countries (LICs) to improve social and health outcomes. PLA represents both a learning philosophy and practical framework that could be applied to a variety of contexts. To date, PLA has not been widely implemented within high-income countries (HICs) to improve health and health-related outcomes. We aim to synthesise the literature currently available by means of systematic review, to form a foundation for future applications of PLA methodology in HICs.

Methods and analysis

Two reviewers will independently search pre-defined terms in the following electronic bibliographic databases: MEDLINE, EMBASE, CINAHL and the Cochrane Library. Search terms will encompass PLA and PDSA (Plan, Do, Study Act) projects, as well as studies using the Triple/ Quadruple Aim model. We will include randomised controlled trials that incorporate online or face-to-face components using PLA/PDSA methodology. Our data will be extracted into a standardised pre-piloted form with subsequent narrative review according to Synthesis without meta-analysis (SWiM) guidelines.

Ethics and dissemination

No ethics approval is required for this study. The results for this study will be submitted for publication in a leading peer-reviewed academic journal in this field. Additionally, a report will be produced for the funders of this review, which can be viewed for free on their website.

For peer review only

Word count: 2576/Max 4000

PROSPERO registration number : CRD42020187978

STRENGTHS AND LIMITATIONS OF THIS STUDY

- Incorporation of multiple participatory research methods to accommodate for the variation in nomenclature describing the intervention
- Broad inclusion criteria to allow for wide heterogeneity of PLA study designs
- Potential low number of RCTs/observational studies for inclusion

- Only includes English language studies from 3 databases

INTRODUCTION

The Participatory learning and action (PLA) approach is one of many methods in the field of participatory research, which also includes Participatory Action Research and Community-Based Participatory Research.[1] In comparison to traditional research methods, the underlying philosophy for these methods is to empower communities to identify their own solutions for issues that affect them, rather than for external stakeholders to enforce a solution on to them.[2,3] The onus for researchers is to seek and include diverse opinions and ensure an open space to discuss these. In doing so, researchers can gain first-hand knowledge from communities, often allowing them to collaborate with marginalised members who had previously low engagement. There are multiple techniques that researchers can use to facilitate this process, including drawing community maps of local resources or landmarks, preference rankings to prioritise issues of the group or Venn diagrams to show the sphere of influence of particular individuals or organisations.[1,3,4]

The PLA group cycle is an iterative process typically led by a facilitator who facilitates the participants through a four-stage cycle of identifying and prioritizing contextual issues, designing strategies to address these issues, putting these strategies into practice and a post-implementation evaluation. It aims to support members to take individual and collective action to solve health problems within their community.[5] Through direct engagement, PLA serves to empower communities to understand the importance of their health and the health of their families and communities, as well as the barriers and facilitators to optimising these health outcomes. Due to its demonstrated efficacy, the World Health Organisation has recommended the implementation of groups practicing PLA in rural low income country settings with reduced access to health services in the context of maternal and newborn health.[6,7]

The current PLA model originated from multiple policies and theories from the last century. In his seminal work ‘Pedagogy of the Oppressed’, Paulo Freire defined that through critical evaluation, the ‘oppressed’ could re-evaluate and improve their context.[8,9] Around the late 1970s, Robert Chambers developed a more participatory research tool, ‘Rapid Rural Appraisal’, as an alternative to more didactic learning methods.[4] This tool later developed into

For peer review only

the PLA cycle, but similar models have also emerged such as the Plan-Do-Study-Act (PDSA) cycle.[10] The PDSA cycle involves planning an intervention, delivering it and examining its effect before making adjustments based on feedback. As such, it has considerable overlap with the PLA model. However, it should be noted that the initial 'planning' stage of PLA involves stakeholders and therefore a greater element of unpredictability. In contrast, PDSA cycles do not usually have the same extent of collaboration in the planning stage. [11]

Within healthcare settings, PDSA cycles have been incorporated into the 'Model for Improvement' to expedite the Quality Improvement (QI) process.[12] QI is integral to the assessment of health services and delivering better-value, higher quality care for patients, especially important in a climate of rising healthcare costs and limited funding.[13] QI methodology can be applied to review a variety of outcomes, including waiting times, patient experience or post-operative complications. A popular framework for measuring healthcare outcomes is the 'Triple Aim' model.[14] Briefly, the three aims are as follows: improving patient experience of healthcare, improving population health and reducing per capita cost of healthcare. Increasingly, an additional fourth aim, to improve the wellbeing of healthcare workers, is integrated to form the 'Quadruple Aim'.[15]

Thus far, PLA cycles have been successfully incorporated in research to improve health outcomes. Prost et al found that women's groups that utilise PLA initiatives have reduced maternal and neonatal mortality in low-income settings (Nepal[16], India[17–19], Bangladesh[20,21] and Malawi[22,23]).[3] The most significant reduction in neonatal mortality (33%) and maternal mortality (49%) occurred in four of the sites that had the highest coverage of groups and participation of at least 30% of pregnant women. The underlying mechanisms are complex.[1,24] Some benefit can be attributed to effects such as behaviour change for hygiene, nutrition and infant care, as well as social support and birth planning. Additionally, the groups worked towards female empowerment and better decision-making to improve access to basic services, encourage safer delivery practices and reduce delays in reaching care. However, women's groups also provide poor, excluded women a voice by building their confidence to make decisions and to reduce the stigma in their lives, while possibly reducing maternal stress.

The focus of PLA health initiatives in low-income countries (LICs) to date has largely been upon maternal and infant nutrition, but has expanded more recently to address both communicable[25] and non-communicable

For peer review only

diseases[8,26]. For example, Tripathy et al set up a PLA women's group for pregnant mothers to reduce neonatal mortality in rural Jharkhand and Odisha in Eastern India.[19] Townsend utilised the PLA methodology to develop projects to enhance peer-based care for HIV positive prisoners in Malaysia.[25] PLA cycles have also been augmented with interventions such as health systems strengthening[22], cash transfers[27] and home counselling[28]. Harris-Fry et al combined PLA women's group with either cash or food transfers to improve maternal diets and nutrition during pregnancy in the Dhanusha and Mahottari districts in Nepal.[29]

Other applications of PLA groups are emerging. Valdez et al. identified that youth PLA groups had a positive impact on young people in terms of substance use prevention.[30] These interventions mainly took place in the United States, with some studies included from Canada and Bosnia Herzegovina. Benefits included greater awareness of drug and alcohol misuse in their community and decreased approval of peer drug use.

The wide variety of applications of PLA groups has contributed to a phenomenon of 'global innovation'. Innovations developed, evaluated and implemented in LICs are adapted and tested within high-income countries (HICs), and are gaining popularity in HICs.[29] An exemplary initiative would be the Partners in Health's 'Prevention and Access to Care and Treatment (PACT) project. Using the home-based medical and social support service used in rural Haiti, PACT was able to increase medical adherence and therefore, positive medical outcomes for HIV patients in Boston, United States.[31] It has since been adopted across cities in the US and globally. Similarly, The Nurture Early for Optimal Nutrition (NEON) study aims to translate the achievements from Bangladesh, India, Malawi, and Nepal noted in Prost et al's work to improve infant feeding and care practices in Tower Hamlets in London.[32] Further, the co-adaptation of community participatory group programmes to support and improve outcomes for parent/carers of children with neurodisability is being co-developed to be ready for piloting in Newham, East London.[33]

To understand this phenomenon, it is also important to acknowledge that different patterns of delivery exist between LICs and HICs. Face-to-face engagements are the norm for PLA as historically their usage has been within LICs, wherein online infrastructure tends to be less well developed. Unsurprisingly, a higher proportion of PLA applications in HICs implement online components, such as online seminars, forums and surveys for data collection.[9,34]

For peer review only

As a proven cost-effective measure with the potential for significant improvement in health outcomes, there is clear applicability of PLA within high-resource settings. Studies to date have variously centred upon the viability and structure of PLA groups in high and low-resource settings, involving multiple populations and contexts.[35,36] At present, no systematic review exists, which synthesises the populations, types of intervention (online or face-to-face) and specific health-related outcomes of PLA groups in HICs.

In this review, we aim to identify the number and types of communities that have engaged in PLA methodology in high-resource settings to date and to analyse any existing documented outcomes of PLA for health and social determinants of health.

METHODS AND ANALYSIS

This protocol was developed in accordance with the Preferred Reporting Items for Systematic Reviews and Meta-Analyses Protocols (PRISMA-P) checklist. This protocol has been registered in PROSPERO (CRD42020187978).

Population, interventions and outcomes

The review will examine published randomised controlled trials (RCTs) and other observational studies in peer-reviewed journals which assess the beneficial effects of PLA groups in social and health outcomes. Interventions can include online or face-to-face groups based in HICs, classified according to the World Bank classification as countries with an income per capita of greater than $12,535.[37] The primary outcome would be any reported improvement related to health or social outcomes after incorporating the PLA model.

Inclusion and exclusion criteria

We will include RCTs and observational studies such as qualitative studies or quality improvement studies in HICs that incorporate online or face-to-face group sessions, which apply the learning and action framework. The review will comprise articles published between 2000 and 2020, to ensure that study interventions are reflective of the current technology and practices. We have decided to exclude any article not written in English, to limit possible risk of misconstruing translated findings from use of online translation services.

Aside from participatory action research, we will incorporate studies that incorporate the PDSA and Triple/ Quadruple aim frameworks.

Search strategy

For peer review only

Two reviewers will independently search pre-defined terms in the following electronic bibliographic databases: MEDLINE, EMBASE, CINAHL and the Cochrane Library. Search terms will encompass PLA and PDSA projects, as well as studies using the Triple/ Quadruple Aim model (Table 1).

Table 1 Proposed terms for search strategy

**Population**	High-income settings filter
**Intervention**	“action learning" or "active learning" or "action cycle" or "learning cycle" or "experiential learning" or "problem-based learning" or “peer support” or “telephone support” or “home visits” “online support group” or “support group” or “participatory group” or “online participatory group” or “participatory learning and action” or “participatory action” or “community participation” or “PDSA” or “PDCA” or “plan-do-study-act” or “plan do study act” or “plan-do-check-act” or “plan do check act” or “women’s group” or “quality improv*” or “quality enhanc*”
**Outcomes**	“triple aim” or “quadruple aim” or “satisfaction” or “patient experience” or “experience of care” or “quality of life” or “health outcome” or “social outcome” or “population health” or “per capita” or “cost” or “cost-effectiveness” or “expenditure” or “cost analysis”

Data analysis and synthesis

Abstracts and articles retrieved from the databases will be imported into the Covidence systematic review management, which automatically detects duplicates and was manually removed.[38] Endnote will be used as reference management software.[39] After removing duplicates using Covidence software, two independent authors will review the search results to identify those meeting the inclusion criteria. The first stage will screen the studies using only the title and abstracts before proceeding to review the full texts. Our preliminary search indicated that the limited literature available on community groups propagating a PLA cycle in high-income countries are to improve specific health or social outcomes. Therefore, we will take care when comparing studies with disparate aims. A flow chart will be created to show an overview of the selection process, including the number of excluded papers and the reasons for exclusion (Figure 1). At any stage, if there are any disagreements between the authors, a third reviewer will be involved to resolve it through discussion.

Figure 1 Proposed Reporting Items for Systematic Reviews flow diagram of the search selection for this systematic review

For peer review only - http://bmjopen.bmj.com/site/about/guidelines.xhtml

Data will be extracted independently by two reviewers using a standardised, pre-piloted form. The extracted information will include: name of the study, study setting, publication date, journal, authors, sample size, study population, participant demographics and baseline characteristics, population inclusion criteria, details of intervention and control, study methodology, recruitment and study completion rates, length of study, follow-up retention percentage, age of participants, outcomes and times of measurement, indicators of acceptability to users, suggested mechanisms of intervention action, RR, mean differences, type of data analysis, information for risk of bias assessment, source of funding and conflict of interest. Any discrepancies will be resolved through discussion or if necessary, a third reviewer. Missing data will be requested by contacting study authors.

Since we anticipate the outcome of selected studies to vary significantly, we will provide a narrative synthesis of the findings from the included studies without conducting a quantitative analysis. The narrative synthesis will be reported according to the SwiM guidelines and will address the methodology of each study, including assessing for risk of bias.[40] Included studies will be grouped according to the type of intervention, target population characteristics, type of outcome and intervention content.

Due to the expected diverse nature of interventions, we will use a standardised metric to represent the result of each outcome. We will calculate risk ratios for dichotomous outcomes and standardised mean differences for continuous outcomes. The results of each study will be summarised and presented in a tabular form. In doing so, we can systematically assess each study result and assess heterogeneity of key study characteristics. If sufficient data is available, we will conduct a subgroup analysis of different types of participants, for example, women or children.

Assessment of risk of bias

Two reviewers will independently assess the risk of bias in included studies by considering the Cochrane Collaboration's tool.[41] We will appraise the following characteristics: random sequence generation, allocation concealment, blinding of participants, personnel and outcome assessment, completeness of outcome data, selective outcome reporting and any other potential sources of bias. The reviewers will assess each domain as low, unclear or high risk as per the tool. Any disparities in the assessment of risk

For peer review only

between the reviewers will be resolved through discussion, involving a third reviewer where necessary.

Patient and Public Involvement

No patient involved

DISCUSSION

Following a preliminary search, a limited number of studies exist describing action learning or similar interventions with patient groups in HICs to improve health or social outcomes [42-56]. PLA is a cost-effective and context specific model which has been shown to improve health outcomes in LICs. Therefore, there exists a need to clarify how the field can advance in its understanding of current documented applications of PLA within HIC settings.

We aim to bridge this gap by conducting a systematic review of the current available evidence on PLA in HICs, in order to understand present implementation and better inform future trials. We anticipate a broad heterogeneity in the data gathered owing to the wide contextuality of PLA and the flexibility of recorded outcomes under the PLA philosophy.

We hope that the findings of our review will result in the following outcomes. First, to establish a benchmark in the field for future reference. Second, the ability to elaborate or clarify existing applications of PLA to replicate observed effects. Third, to identify health outcomes that have not previously received attention with regards to implementation of PLA methodology in any setting. Fourth, to examine which PLA modalities relate to which health outcomes and evaluate the significance of these.

We also hope that our review will inform the designing and planning phases of future trials. This could be through guiding possible effect and therefore sample sizes, selecting appropriate outcomes and suitable follow-up periods. As it has within several LICs, implementation of PLA could pave the way to influencing healthcare policies and inducing systemic changes to health provision. In summary, a comprehensive review of current literature will enable prioritisation of future research directions.

ETHICS AND DISSEMINATION

For peer review only

No ethics approval is required for this study. The results for this study will be submitted for publication in a leading peer-reviewed academic journal in this field. Additionally, a report will be produced for the funders of this review, which can be viewed for free on their website.

FIGURE AND TABLE LEGENDS

Table 1 - Proposed terms for search strategy
Figure 1 - Proposed Reporting Items for Systematic Reviews flow diagram of the search selection for this systematic review

References

1. de Brún T, O'Reilly-De Brún M, Van Weel-Baumgarten E, *et al.* Using Participatory Learning & Action (PLA) research techniques for inter-stakeholder dialogue in primary healthcare: an analysis of stakeholders' experiences. *Research Involvement and Engagement* 2017;**3**:28. doi:10.1186/s40900-017-0077-8
2. Intrac. Participatory Learning and Action (PLA). 2017. https://www.intrac.org/wpcms/wp-content/uploads/2017/01/Participatory-learning-and-action.pdf (accessed 4 Nov 2020).
3. Chambers R. Relaxed and Participatory Appraisal: notes on practical approaches and methods for participants in PRA/PLA-related familiarisation workshops. Participation at Resource Centre, Institute of Development Studies, Brighton, UK. 2002.http://eepsea.org/uploads/user-S/11491553671Reader_1_PRA_notes.pdf (accessed 8 Nov 2020).
4. Appel K, Buckingham E, Jodoin K, *et al.* Participatory Learning and Action Toolkit: For Application in BSR's Global Programs. *Bsr* 2012;:48.https://herproject.org/files/toolkits/HERproject-Participatory-Learning.pdf (accessed 4 Nov 2020).
5. Seward N, Neuman M, Colbourn T, *et al.* Effects of women's groups practising participatory learning and action on preventive and care-seeking behaviours to reduce neonatal mortality: A meta-analysis of cluster-randomised trials. *PLoS medicine* 2017;**14**:e1002467. doi:10.1371/journal.pmed.1002467
6. World Health Organization. WHO recommendation on facilitated participatory learning and action cycles with women`s groups during pregnancy. WHO Reproductive Health Library. 2016.https://extranet.who.int/rhl/topics/improving-health-system-performance/implementation-strategies/who-recommendation-facilitated-participatory-learning-and-action-cycles-womens-groups-during (accessed 21 May 2020).
7. Prost A, Colbourn T, Seward N, *et al.* Women's groups practising participatory learning and action to improve maternal and newborn health in low-resource settings: A systematic review and meta-analysis. *The Lancet* 2013;**381**:1736–46. doi:10.1016/S0140-6736(13)60685-6
8. Morrison J, Akter K, Jennings HM, *et al.* Participatory learning and action to address type 2 diabetes in rural Bangladesh: A qualitative process evaluation. *BMC Endocrine Disorders* 2019;**19**. doi:10.1186/s12902-019-0447-3
9. Cueva K, Cueva M, Revels L, *et al.* A Framework for Culturally Relevant Online

For peer review only

Learning: Lessons from Alaska's Tribal Health Workers. *Journal of Cancer Education* 2019;**34**:647–53. doi:10.1007/s13187-018-1350-8
10. Deming W. *The New Economics for Industry, Government, Education (2nd edition) | IHI - Institute for Healthcare Improvement*. Cambridge, Massachusetts: : The MIT Press 2000. http://www.ihi.org/resources/Pages/Publications/NewEconomicsforIndustryGovernmentEducation.aspx (accessed 23 Aug 2020).
11. Institute of Healthcare Improvement. Science of Improvement: How to Improve. Institute of Healthcare Improvement. 2009.http://www.ihi.org/resources/Pages/HowtoImprove/ScienceofImprovementHowtoImprove.aspx (accessed 23 Aug 2020).
12. Taylor, M., McNicholas, C., Nicolay, C., Darzi, A., Bell, D. and Reed, J., 2013. Systematic review of the application of the plan–do–study–act method to improve quality in healthcare. *BMJ Quality & Safety*, 23(4), pp.290-298.
13. Jones B, Vaux E, Olsson-Brown A. How to get started in quality improvement. *BMJ (Online)* 2019;**364**. doi:10.1136/bmj.k5437
14. Berwick DM, Nolan TW, Whittington J. The triple aim: Care, health, and cost. *Health Affairs* 2008;**27**:759–69. doi:10.1377/hlthaff.27.3.759
15. Sikka R, Morath JM, Leape L. The quadruple aim: Care, health, cost and meaning in work. *BMJ Quality and Safety* 2015;**24**:608–10. doi:10.1136/bmjqs-2015-004160
16. Manandhar PDS, Osrin D, Prasad Shrestha B, *et al.* Effect of a participatory intervention with women's groups on birth outcomes in Nepal: Cluster-randomised controlled trial. *Lancet* 2004;**364**:970–9. doi:10.1016/S0140-6736(04)17021-9
17. Tripathy P, Nair N, Barnett S, *et al.* Effect of a participatory intervention with women's groups on birth outcomes and maternal depression in Jharkhand and Orissa, India: a cluster-randomised controlled trial. *The Lancet* 2010;**375**:1182–92. doi:10.1016/S0140-6736(09)62042-0
18. More NS, Bapat U, Das S, *et al.* Community mobilization in Mumbai slums to improve perinatal care and outcomes: A cluster randomized controlled trial. *PLoS Medicine* 2012;**9**:e1001257. doi:10.1371/journal.pmed.1001257
19. Tripathy P, Nair N, Sinha R, *et al.* Effect of participatory women's groups facilitated by Accredited Social Health Activists on birth outcomes in rural eastern India: A cluster-randomised controlled trial. *The Lancet Global Health* 2016;**4**:e119–28. doi:10.1016/S2214-109X(15)00287-9
20. Azad K, Barnett S, Banerjee B, *et al.* Effect of scaling up women's groups on birth outcomes in three rural districts in Bangladesh: a cluster-randomised controlled trial. *The Lancet* 2010;**375**:1193–202. doi:10.1016/S0140-6736(10)60142-0
21. Fottrell E, Azad K, Kuddus A, *et al.* The effect of increased coverage of participatory women's groups on neonatal mortality in Bangladesh: A cluster randomized trial. *JAMA Pediatrics* 2013;**167**:816–25. doi:10.1001/jamapediatrics.2013.2534
22. Lewycka S, Mwansambo C, Kazembe P, *et al.* A cluster randomised controlled trial of the community effectiveness of two interventions in rural Malawi to improve health care and to reduce maternal, newborn and infant mortality. *Trials* 2010;**11**. doi:10.1186/1745-6215-11-88
23. Colbourn T, Nambiar B, Bondo A, *et al.* Effects of quality improvement in health facilities and community mobilization through women's groups on maternal, neonatal and perinatal mortality in three districts of Malawi: MaiKhanda, a cluster

For peer review only

randomized controlled effectiveness trial. *International Health* 2013;**5**:180–95. doi:10.1093/inthealth/iht011
24. Gram L, Skordis-Worrall J, Manandhar DS, *et al.* The long-term impact of community mobilisation through participatory women's groups on women's agency in the household: A follow-up study to the Makwanpur trial. *PLoS ONE* 2018;**13**. doi:10.1371/journal.pone.0197426
25. Townsend D. Prisoners with HIV/AIDS: A participatory learning and action initiative in Malaysia. *Tropical Doctor* 2001;**31**:8–10. doi:10.1177/004947550103100103
26. Fottrell E, Ahmed N, Morrison J, *et al.* Community groups or mobile phone messaging to prevent and control type 2 diabetes and intermediate hyperglycaemia in Bangladesh (DMagic): a cluster-randomised controlled trial. *The Lancet Diabetes and Endocrinology* 2019;**7**:200–12. doi:10.1016/S2213-8587(19)30001-4
27. Harris-Fry HA, Paudel P, Harrisson T, *et al.* Participatory women's groups with cash transfers can increase dietary diversity and micronutrient adequacy during pregnancy, whereas women's groups with food transfers can increase equity in intrahousehold energy allocation. *Journal of Nutrition* 2018;**148**:1472–83. doi:10.1093/jn/nxy109
28. Nair N, Tripathy P, Sachdev HS, *et al.* Effect of participatory women's groups and counselling through home visits on children's linear growth in rural eastern India (CARING trial): a cluster-randomised controlled trial. *The Lancet Global Health* 2017;**5**:e1004–16. doi:10.1016/S2214-109X(17)30339-X
29. DePasse JW, Lee PT. A model for 'reverse innovation' in health care. *Globalization and Health* 2013;**9**:40. doi:10.1186/1744-8603-9-40
30. Valdez ES, Skobic I, Valdez L, *et al.* Youth Participatory Action Research for Youth Substance Use Prevention: A Systematic Review. *Substance Use and Misuse* 2020;**55**:314–28. doi:10.1080/10826084.2019.1668014
31. Behforouz HL, Farmer PE, Mukherjee JS. From direcdy observed therapy to Accompagnateurs: Enhancing AIDS treatment outcomes in Haiti and in Boston. *Clinical Infectious Diseases* 2004;**38**:S429–36. doi:10.1086/421408
32. Lakhanpaul M, Benton L, Lloyd-Houldey O, *et al.* Nurture Early for Optimal Nutrition (NEON) programme: qualitative study of drivers of infant feeding and care practices in a British-Bangladeshi population. *BMJ open* 2020;**10**:e035347. doi:10.1136/bmjopen-2019-035347
33. Skopec M, Issa H, Harris M. Delivering cost effective healthcare through reverse innovation. *The BMJ* 2019;**367**:l6205. doi:10.1136/bmj.l6205
34. Ambrose M, Murray L, Handoyo NE, *et al.* Learning global health: a pilot study of an online collaborative intercultural peer group activity involving medical students in Australia and Indonesia. BMC Medical Education 2017;17:1–11. doi:10.1186/s12909-016-0851-6
35. Anyon Y, Bender K, Kennedy H, et al. A Systematic Review of Youth Participatory Action Research (YPAR) in the United States: Methodologies, Youth Outcomes, and Future Directions. Health Education and Behavior 2018;45:865–78. doi:10.1177/1090198118769357
36. Backhouse T, Kenkmann A, Lane K, et al. Older Care-Home residents as collaborators or advisors in research: A systematic review. Age and Ageing 2016;45:337–45. doi:10.1093/ageing/afv201
37. World Bank Data Team. New country classifications by income level: 2019-2020.

For peer review only

World Bank Blogs. 2019.https://blogs.worldbank.org/opendata/new-country-classifications-income-level-2019-2020 (accessed 22 June 2020).
38. Covidence systematic review software, Veritas Health Innovation, Melbourne, Australia. Available at www.covidence.org
39. Endnote reference management programme, Clarivate, Available at https://endnote.com.
40. Campbell M, McKenzie JE, Sowden A, et al. Synthesis without meta-analysis (SWiM) in systematic reviews: Reporting guideline. The BMJ 2020;368. doi:10.1136/bmj.l6890
41. Sterne JAC, Savović J, Page MJ, et al. RoB 2: A revised tool for assessing risk of bias in randomised trials. The BMJ 2019;366. doi:10.1136/bmj.l4898
42. Anderson RM, Funnell MM, Nwankwo R, Gillard ML, Oh M, Fitzgerald JT. Evaluating a problem-based empowerment program for African Americans with diabetes: results of a randomized controlled trial. Ethnicity & disease [Internet]. 2005; 15(4):[671-8 pp.]. Available from: http://onlinelibrary.wiley.com/o/cochrane/clcentral/articles/745/CN-00552745/frame.html.
43. Drysdale J, Casey J, Porter-Armstrong A. Effectiveness of training on the community skills of children with intellectual disabilities. Scandinavian Journal of Occupational Therapy. 2008;15(4):247-55. PubMed PMID: 18855229.
44. Jacobsson LR, Friedrichsen M, Goransson A, Hallert C. Impact of an active patient education program on gastrointestinal symptoms in women with celiac disease following a gluten-free diet: a randomized controlled trial. Gastroenterology Nursing. 2012;35(3):200-6. PubMed PMID: 22647800.
45. Kamwendo K, Tingström P, Bergdahl B, Svensson E. Effect of problem-based learning on stages of change for exercise behaviour in patients with coronary artery disease. Physiotherapy research international : the journal for researchers and clinicians in physical therapy [Internet]. 2004; 9(1):[24-32 pp.]. Available from: http://onlinelibrary.wiley.com/o/cochrane/clcentral/articles/914/CN-00467914/frame.html.
46. Ringsberg KC, Timpka T. Clinical health education for patients with asthma-like symptoms but negative asthma tests. Allergy [Internet]. 2001; 56(11):[1049-54 pp.]. Available from: http://onlinelibrary.wiley.com/o/cochrane/clcentral/articles/298/CN-00375298/frame.html.
47. Tingström PR, Ekelund U, Kamwendo K, Bergdahl B. Effects of a problem-based learning rehabilitation program on physical activity in patients with coronary artery disease. Journal of cardiopulmonary rehabilitation [Internet]. 2006; 26(1):[32-8 pp.]. Available from: http://onlinelibrary.wiley.com/o/cochrane/clcentral/articles/237/CN-00564237/frame.html.
48. Naik AD, Teal CR, Rodriguez E, Haidet P. Knowing the ABCs: a comparative effectiveness study of two methods of diabetes education. Patient education and counseling [Internet]. 2011; 85(3):[383-9 pp.]. Available from: http://onlinelibrary.wiley.com/o/cochrane/clcentral/articles/479/CN-00837479/frame.html.
49. Nunez M, Nunez E, Yoldi C, Quinto L, Hernandez Ma V, Munoz-Gomez J. Health-

For peer review only

related quality of life in rheumatoid arthritis: Therapeutic education plus pharmacological treatment versus pharmacological treatment only. Rheumatology International. 2006 June;26(8):752-7. PubMed PMID: 2006231426.
50. Nunez M, Nunez E, Segur JM, Macule F, Quinto L, Hernandez MV, et al. The effect of an educational program to improve health-related quality of life in patients with osteoarthritis on waiting list for total knee replacement: A randomized study. Osteoarthritis and Cartilage. 2006 March;14(3):279-85. PubMed PMID: 2006107634.
51. Pichert JW, Snyder GM, Kinzer CK, Boswell EJ. Problem solving anchored instruction about sick days for adolescents with diabetes. Patient Education & Counseling. 1994;23(2):115-24. PubMed PMID: 21207910.
52. Rosen L, Zucker D, Brody D, Engelhard D, Manor O. The effect of a handwashing intervention on preschool educator beliefs, attitudes, knowledge and self-efficacy. Health education research. 2009;24(4):686-98. PubMed PMID: 19318523.
53. Sarkadi A, Rosenqvist U. Experience-based group education in Type 2 diabetes: a randomised controlled trial. Patient Education & Counseling. 2004;53(3):291-8. PubMed PMID: 15186866.
54. Wong MCS, Lau TCM, Lee A. The Impact of Leadership Programme on Self-Esteem and Self-Efficacy in School: A Randomized Controlled Trial. PLoS ONE. 2012 18 Dec;7(12). PubMed PMID: 2012742201.
55. Ring Jacobsson L, Friedrichsen M, Goransson A, Hallert C. Does a Coeliac School increase psychological well-being in women suffering from coeliac disease, living on a gluten-free diet? Journal of Clinical Nursing. 2012;21(5-6):766-75. PubMed PMID: 22039932.
56. Reid MJ, Webster-Stratton C, Hammond M. Enhancing a classroom social competence and problem-solving curriculum by offering parent training to families of moderate- to high-risk elementary school children. Journal of Clinical Child & Adolescent Psychology. 2007;36(4):605-20. PubMed PMID: 18088218.

For peer review only

Authors' contributions:

MH, SA, LM, ML conceived the original concept of the study and designed the research methodology. SA, AK, and FM carried out the registration and drafted the manuscript. MH, ML, LM, EW, CS, JM, and AC validated the study and revised the manuscript critically for important intellectual content. SA, AK, FM contributed to the manuscript writing, edited the final manuscript and prepared it for submission. MH, SA had primary responsibility for the final content. All authors read and contributed to reviewing the paper, the designing of the manuscript, and the approval of the final manuscript.

Funding statement:

Logan Manikam and Shereen Al Laham are funded via a National Institute for Health Research (NIHR) Advanced Fellowship (Ref: NIHR300020) to undertake the pilot randomised controlled trial of the NEON study in East London. Dr Michelle Heys is funded by Barts Charity to

undertake G2KCP study. Prof Monica Lakhanpaul was funded by the NIHR Collaboration for Leadership in Applied Health Research and Care (CLAHRC) North Thames.

Competing interests statement.

There is no conflict of interest to declare

For peer review only

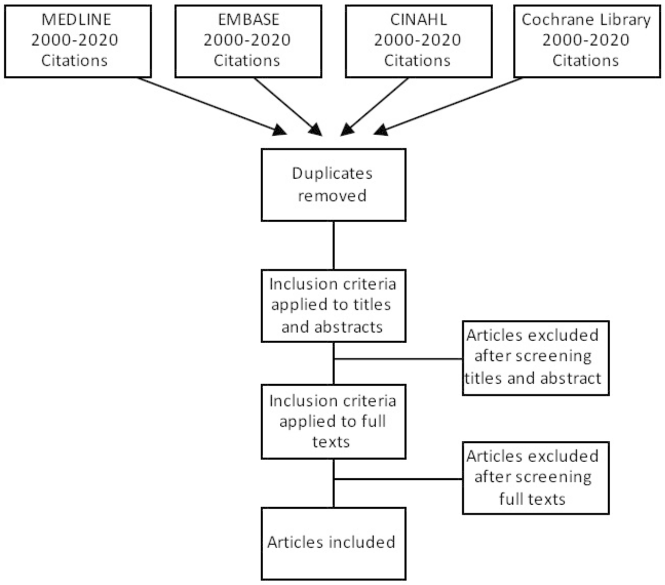

Preferred Reporting Items for Systematic Reviews- flow diagram of the search selection for this systematic review.

Reporting checklist for protocol of a systematic review and meta analysis.

Based on the PRISMA-P guidelines.

Instructions to authors

Complete this checklist by entering the page numbers from your manuscript where readers will find each of the items listed below.

Your article may not currently address all the items on the checklist. Please modify your text to include the missing information. If you are certain that an item does not apply, please write "n/a" and provide a short explanation.

Upload your completed checklist as an extra file when you submit to a journal.

In your methods section, say that you used the PRISMA-Preporting guidelines, and cite them as:

Moher D, Shamseer L, Clarke M, Ghersi D, Liberati A, Petticrew M, Shekelle P, Stewart LA. Preferred Reporting Items for Systematic Review and Meta-Analysis Protocols (PRISMA-P) 2015 statement. Syst Rev. 2015;4(1):1.

For peer review only

		Reporting Item	Page Number
**Title**			
Identification	#1a	Identify the report as a protocol of a systematic review	1
Update	#1b	If the protocol is for an update of a previous systematic review, identify as such	N/A - not for an update of a previous systematic review
**Registration**			
	#2	If registered, provide the name of the registry (such as PROSPERO) and registration number	2
**Authors**			
Contact	#3a	Provide name, institutional affiliation, e-mail address of all protocol authors; provide physical mailing address of corresponding author	1

Contribution	#3b	Describe contributions of protocol authors and identify the guarantor of the review	15
**Amendments**			
	#4	If the protocol represents an amendment of a previously completed or published protocol, identify as such and list changes; otherwise, state plan for documenting important protocol amendments	N/A - not an amendment of a previous protocol
**Support**			
Sources	#5a	Indicate sources of financial or other support for the review	15
Sponsor	#5b	Provide name for the review funder and / or sponsor	15
Role of sponsor or funder	#5c	Describe roles of funder(s), sponsor(s), and / or institution(s), if any, in developing the protocol	15
**Introduction**			
Rationale	#6	Describe the rationale for the review in the context of what is already known	3-6
Objectives	#7	Provide an explicit statement of the question(s) the review will address with reference to participants, interventions, comparators, and outcomes (PICO)	6,7
**Methods**			
Eligibility criteria	#8	Specify the study characteristics (such as PICO, study design, setting, time frame) and report characteristics (such as years considered, language, publication status) to be used as criteria for eligibility for the review	7
Information sources	#9	Describe all intended information sources (such as electronic databases, contact with study authors, trial registers or other grey literature sources) with planned dates of coverage	7
Search strategy	#10	Present draft of search strategy to be used for at least one electronic database, including planned limits, such that it could be repeated	7,8

For peer review only

Study records - data management	#11a	Describe the mechanism(s) that will be used to manage records and data throughout the review	7,8
Study records - selection process	#11b	State the process that will be used for selecting studies (such as two independent reviewers) through each phase of the review (that is, screening, eligibility and inclusion in meta-analysis)	7,8
Study records - data collection process	#11c	Describe planned method of extracting data from reports (such as piloting forms, done independently, in duplicate), any processes for obtaining and confirming data from investigators	7,8
Data items	#12	List and define all variables for which data will be sought (such as PICO items, funding sources), any pre-planned data assumptions and simplifications	6-8
Outcomes and prioritization	#13	List and define all outcomes for which data will be sought, including prioritization of main and additional outcomes, with rationale	6-8
Risk of bias in individual studies	#14	Describe anticipated methods for assessing risk of bias of individual studies, including whether this will be done at the outcome or study level, or both; state how this information will be used in data synthesis	9
Data synthesis	#15a	Describe criteria under which study data will be quantitatively synthesised	N/A - quantitative synthesis
Data synthesis	#15b	If data are appropriate for quantitative synthesis, describe planned summary measures, methods of handling data and methods of combining data from studies, including any planned exploration of consistency (such as I2, Kendall's τ)	9
Data synthesis	#15c	Describe any proposed additional analyses (such as sensitivity or subgroup analyses, meta-regression)	9
Data synthesis	#15d	If quantitative synthesis is not appropriate, describe the type of summary planned	9
Meta-bias(es)	#16	Specify any planned assessment of meta-bias(es) (such as publication bias across studies, selective reporting within studies)	9

For peer review only

Confidence in cumulative evidence	#17	Describe how the strength of the body of evidence will be assessed (such as GRADE)	9

Notes:

- 1b: N/A - not for an update of a previous systematic review
- 4: N/A - not an amendment of a previous protocol
- 15a: N/A - quantitative synthesis The PRISMA-P elaboration and explanation paper is distributed under the terms of the Creative Commons Attribution License CC-BY. This checklist was completed on 28. February 2021 using https://www.goodreports.org/, a tool made by the EQUATOR Network in collaboration with Penelope.ai

For peer review only
